# Hysteroscopy as a Therapeutic Tool: A Vision to Spare the Uterus in Premenopausal Abnormal Uterine Bleeding (AUB)/Heavy Menstrual Bleeding (HMB), an Update

**DOI:** 10.7759/cureus.47877

**Published:** 2023-10-28

**Authors:** Neema Acharya, Preeti Mishra, Shazia Mohammad, Megha Karnik, Shaikh Muneeba, Rinkle Gemnani, Keyur Saboo, Samarth Shukla, Sourya Acharya

**Affiliations:** 1 Department of Obstetrics and Gynaecology, Jawaharlal Nehru Medical College, Datta Meghe Institute of Higher Education and Research, Wardha, IND; 2 Department of Pathology, Jawaharlal Nehru Medical College, Datta Meghe Institute of Higher Education and Research, Wardha, IND; 3 Department of Medicine, Jawaharlal Nehru Medical College, Datta Meghe Institute of Higher Education and Research, Wardha, IND

**Keywords:** hysterectomy, quality of life (qol), review article, hysteroscopy, hmb, aub

## Abstract

Heavy menstrual bleeding (HMB) or abnormal uterine bleeding (AUB) is a common cause of gynecological complaints in perimenopausal women. The treatment chosen by most of the women having HMB/AUB in the perimenopausal age group when medical therapy fails is hysterectomy, which unfortunately has significant issues related to safety and long-term quality of life apart from being a burden on the health care system and cost to patients. Minimal access surgical techniques like hysteroscopic targeted therapies are available which are conservative and spare the uterus and major surgery and its complications and sequelae. Hysteroscopic management of HMB is a minimally invasive and targeted approach in diagnosing and treating the pathological lesions causing the symptoms and not radical like hysterectomy which has more chances of occurrence of adverse events both intra and postoperatively. In terms of health-related quality of life, women suffering from HMB who underwent a hysteroscopic conservative approach had better scores when compared to those with hysterectomy.

The present review aims to review the evidence generated to compare the two surgical modalities, hysteroscopic targeted therapy and hysterectomy, in terms of their effectiveness, safety, and effect on the quality of life of these perimenopausal women having HMB/AUB.

## Introduction and background

One of the most important symptoms with which women seek consultation in gynecology is abnormal uterine bleeding (AUB) or heavy menstrual bleeding (HMB). The prevalence of AUB/HMB is reported between 11-13% in all age groups of women increasing to as high as 25 to 30% in those aged 36-40 years [1].

An update in September 2021 by the International Federation of Gynaecology and Obstetrics (FIGO) mentions that prevalence rates of HMB can vary widely according to demography, the definition used for HMB, and other factors. It is estimated that almost 20% of women suffer from AUB/HMB during childbearing and perimenopausal age groups [2].

The reported incidence of HMB/AUB in India is as high as 17 to 30% among gynecologic consultations [1]. Abnormal uterine bleeding is a common cause of perimenopausal age group women’s suffering and it also impairs quality of life (QoL) significantly.

HMB apart from affecting the overall quality of life in terms of physical, mental, and sexual health increases a woman’s risk to undergo surgical procedures like hysterectomy. Unfortunately, hysterectomy is still the most common gynecologic surgery, and most of the time it is done for benign gynecologic diseases and not as a lifesaving procedure. Almost 40 to 50% of women having hysterectomy for HMB/AUB have normal-sized uterus without significant pelvic pathology. In up to 40 to 60% of women endometrial polyp or small submucous fibroid is the underlying pathology that can be removed specifically.

With the advances and expertise in minimally invasive and uterus-sparing procedures done with hysteroscopy in gynecological practice, radical procedures like hysterectomy and associated complications could be avoided in most of the cases of HMB/AUB. Minimally invasive hysteroscopic surgical procedures (e.g., hysteroscopic polypectomy, transcervical resection of the endometrium (TCRE), hysteroscopic myomectomy) aim to give targeted therapy and be conservative in treatment. Apart from TCRE, hysteroscopic polypectomy and myomectomy are targeted surgeries that can completely relieve menstrual symptoms associated with them.

First-line therapy for AUB/HMB is most of the time chosen as a medical method but it is ineffective in some patients. Therefore both the health care providers and patients opt for a hysterectomy as a foolproof or definitive method to cure abnormalities of menstrual bleeding. Though effective, it is costly and is associated with risks of anesthesia and surgery.

Since the last three decades, the approach to benign diseases has been moving towards conservative modalities, especially with the advent of minimally invasive hysteroscopy. It also serves to target therapy and conserve the uterus pelvic anatomy thereby improving the quality of life of these women. Hysteroscopic procedures are less invasive, offer targeted therapy, and preserve the uterus and normal pelvic anatomy and function.

There is a need for a change in the mindset of women and many treating gynecologists toward management options. Women should be made more aware of the availability of uterus-sparing therapeutic modalities that can relieve their primary symptoms and have both short-term and long-term benefits of conserving the uterus on quality of life. Better evidence generation and characterization of comparison of conservative targeted approach by hysteroscopic management of HMB/AUB with hysterectomy will help in making informed decisions and counseling these women.

The present review article aims to assess and compare the uterus-sparing (conserving uterus and avoiding hysterectomy) conservative modality of hysteroscopic therapies with traditional hysterectomy in terms of effectiveness, safety, and change in the quality of life as a therapeutic surgical modality for women suffering from AUB/HMB.

Aim

We aim to review the study and compare the effectiveness in relieving menstrual complaints, safety, and effect on postoperative quality of life of two modalities, uterus-sparing hysteroscopic surgical therapy and hysterectomy for the treatment of HMB/AUB in the premenopausal age group.

Search methods for identification of studies

**Figure 1:**
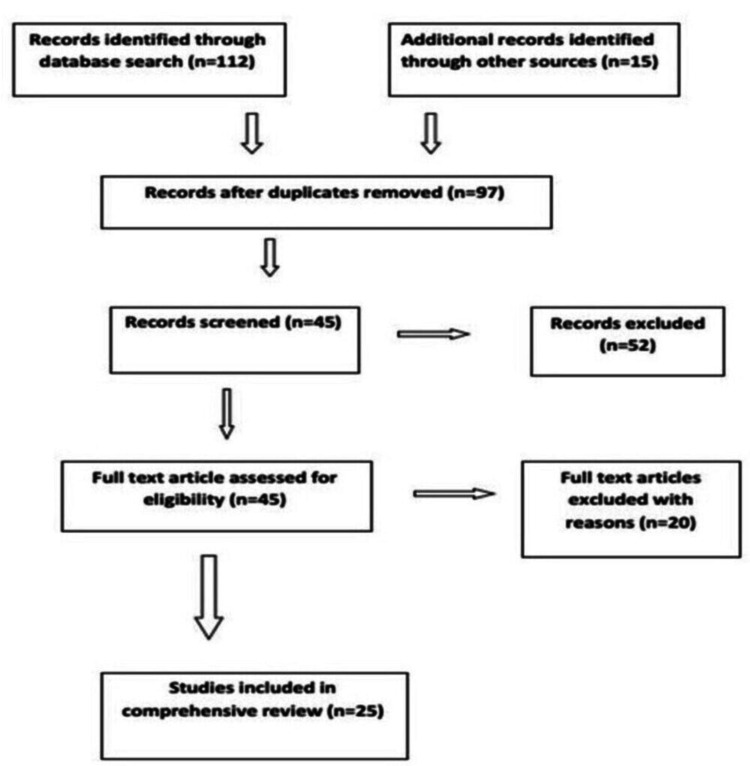
Search strategy applied in the review.

We searched the electronic databases and bibliographies of the retrieved articles of original articles and review articles along with analysis of their references. Related publications of conference proceedings and journals were also analyzed. Medical Subject Headings (MeSH) were used to search individual databases. MeSH terms including all subheadings and keywords we used were hysteroscopy, endometrial polyps, abnormal uterine bleeding, endometrial polyps, hysteroscopic polypectomy, submucous myoma, hysteroscopic myomectomy, hysterectomy, endometrial resection, and health-related quality of life. Figure 1 shows the search strategy applied in this review.

## Review

Sociodemography

Almost 10%-30% of women suffer from HMB/AUB in the perimenopausal age group [3]. Goldstein and Lumsden in their study reported that 20% of gynecological consultations are for HMB/AUB in premenopausal women while it increases to 25% for those who undergo surgical interventions perimenopausal age group [4]. Whitaker and Critchley reported that one-third of all outpatient gynecologic visits in reproductive years and almost 69% in perimenopausal group women are related to heavy or abnormal uterine bleeding [5]. Ontario Health Technology Assessment Series published that for Ontario, it was estimated that about 15% to 20% of women over 30 years have HMB [6].

In a study by Acharya et al. (2019), 42% (148 out of 354) of women having HMB/AUB were less than 40 years of age depicting a significant number of women presenting with AUB/HMB are younger and at risk of undergoing hysterectomy at age less than 40 years. Women opt for and have the risk of undergoing hysterectomy at a young age as low as 32 years and spend the rest of life at risk of sequelae of early menopause. In the hysterectomy group, the lowest age seen was 31 years [1].

Diagnosis

A study comparing transvaginal sonography (TVS) and hysteroscopy in the assessment of HMB/AUB by Goyal et al. showed that in 57% of patients, endometrium and cavity were normal on TVS while myomas polyps and thick endometrial echo complex were found in the rest of the cases [7].

A study done by Bingol et al. reported polypoid lesions led to the diagnosis while hyperplasia was the second most common cause among their study population consisting of 137 women having abnormal uterine bleeding [8].

In their study, Selvanathan et al. analyzed the histopathological diagnosis of AUB/HMB. Twenty-three percent of women had normal pelvic ultrasound findings, 39.33% as thickened endometrium, 16.29% as hyperplasia + polyp, 22.36% as hyperplasia, and 8.99% as fibroid while it was 31.25%, 36.36%, 11.93%, 15.34%, and 5.11% respectively in hysterectomy group. Most of the patients had a suspicion of polyp or hyperplasia [1].

They have further elaborated on the relationship between pelvic ultrasonography (USG) and hysteroscopy findings in the hysteroscopy group. Out of 42 cases reported as normal by USG, 28 had normal hysteroscopy findings while 14 had endometrial polyps. Out of 70 cases suggestive of thickened endometrium by USG, 55 had a polyp, six had hyperplasia + polyp and nine had normal findings. Out of 31 cases suggestive of hyperplasia with polyp, four had only hyperplasia, 18 had hyperplasia with polyp and two had only polyp. Out of 22 cases of hyperplasia, eight had normal findings, nine had polyps and four had hyperplasia. All 13 cases suggestive of fibroid were fibroid on hysteroscopy. This suggests that a direct look inside the uterus gives a definitive diagnosis and treatment could be discussed and targeted. In this study, there was no case of atrophic endometrium as most of our patients were younger (mean age around 40 years) in the age group [1].

Abid et al. conducted a study for analysis of the clinical spectrum and histopathological diagnosis of AUB/HMB. The most common finding was a normal menstrual pattern (34%), followed by anovulatory (27%), benign polyp (14%), endometritis (12%), atrophic endometrium (6%), endometrial hyperplasia (5%), and endometrial carcinoma (2%). They concluded most of the HMB have organic benign pathology and these can be managed by conservative modalities [9].

Effectiveness of two surgical modalities

A pictorial blood assessment chart (PBAC) is a tool used for assessing menstrual blood loss. A PBAC score of 100 or more is taken as heavy bleeding [10]. Following is a review of the effectiveness of hysteroscopic surgical therapy used to treat HMB/AUB in terms of improvement of blood loss and menstrual pattern.

Improvement in PBAC Score

In a study done by Selvanathan et al., the mean preoperative (PBAC) score in the hysteroscopy group was 251.073 (SD of 75.41) while in the hysterectomy group, it was 252.108 (SD of 74.98). There was a significant change in PBAC score and improvement in blood loss in hysteroscopically treated women. At six months postoperatively, it was 70.06 (SD of 38.06) with a mean change in PBAC score of 181.6 with a p-value of 0.000 which is significant (CI of 149.53-172.25). At one year follow-up, the mean PBAC score further reduced to 65.04469 (23.07) with a p-value of 0.000 which is significant depicting that at one year the score remained almost the same and did not deteriorate. The results suggest that women have improved in menstrual bleeding amount and pattern gradually over one year. These points should be considered and explained to women for better acceptance of treatment modalities [1].

A Cochrane systematic review by Fergusson et al. on endometrial resection and ablation versus hysterectomy for heavy menstrual bleeding concluded that conservative endometrial resection and ablation approach was at par with hysterectomy in achieving relief at the end of one, two and four years [11].

Effect on the Menstrual Pattern

Abbott et al. in their study analyzed the effectiveness of endometrial ablation in the treatment of abnormal uterine bleeding. They reported conservative modality is at par in controlling the bleeding amount and pattern in women with AUB and patient satisfaction. In their study, amenorrhea, hypomenorrhea, eumenorrhea and menorrhagia rates for the ablation at 12 months were 16/37 (43%), 11/18 (61%) vs. 10/37 (27%), and 5/18 (27%) [12].

Cochrane systematic review by Lethaby et al. on endometrial resection and ablation methods for the treatment of HMB reports following rates of amenorrhea on two methods of endometrial resection. The amenorrhoea rate was comparable in both groups at the end of one year [13].

Matteson et al. in their systematic review studied seven studies and reported their effect on menstrual bleeding outcomes. The number of women who achieved amenorrhea ranged from 13-64%. Though the amenorrhea rate was 100%, there was one case who had persistent vaginal vault bleeding after a hysterectomy too [14].

Stepniewska et al. studied and compared the long-term effects of another uterine-sparing treatment of adenomyosis with radiofrequency thermal ablation (RFA) in 60 patients. Eight patients underwent hysterectomy. They concluded that RFA can avoid hysterectomy in most of the cases of adenomyosis [15].

Piecak and Milart studied the outcome of hysteroscopic myomectomy. They suggested that most of the submucous myoma can be removed hysteroscopically as a single-step surgery with proper selection of cases with 70 to 99% of women getting symptom-free post-procedure. The success rate is inversely proportional to the age of the woman [16].

Agdi and Tulandi studied the endoscopic management of uterine fibroids. They concluded hysteroscopic myomectomy is an effective method to treat submucous fibroids [17]. Fibroids may recur even after hysteroscopic myomectomy specially in reproductive years as observed in a study by Selvanathan et al. [1]. In their study cases who had recurrence and persistent menorrhagia, both cases were below 40 years of age.

Camanni et al. mention in their study that submucous myoma with a diameter of 6 cm or less is treated more effectively than those of larger size. For larger sizes, two sittings may be needed [18]. Grading myomas as per the LASMAR scoring system allows scoring the fibroid in terms of the need for one or two sittings of surgery and predicting surgical safety [19]. Donnez et al. reported that the failure of hysteroscopic treatment correlated to improper selection of cases, inadequate resection, and coexisting adenomyosis [20]. Van Dongen et al. recommended complete resection of fibroid for relief of symptoms [21]. In a study done by Selvanathan et al., two patients who had persistent menorrhagia were less than 40 years of age. They were found to have a normal uterine cavity with thickened endometrium on ultrasound. This thickening of endometrium could be because of the inherent hyper estrogenic status associated with fibroid. Abdollahi Fard et al. studied hysteroscopy for the treatment of HMB/AUB. Hysteroscopy was successful in 73.5% of the bleeding group [22].

In a study done by Selvanathan et al., dysmenorrhea was seen as an associated symptom of AUB/HMB in 28 out of 178 (15.73%) cases in the hysteroscopy group and 34 out of 176 (19.31%) in the hysterectomy group. All but two women out of 28 (92.2%) hysteroscopy group were relieved of dysmenorrhea.

Adverse Events and Safety

Following is the literature review of the comparison of safety and adverse events when hysteroscopy and hysterectomy are used as treatment modalities for AUB.

Cochrane systematic review by Fergusson et al. compared hysteroscopic management versus hysterectomy as a treatment modality for HMB/AUB. Hysteroscopic management was superior in terms of effectiveness and safety when compared to hysterectomy [11].

A quality-sponsored randomized study of endometrial ablation versus hysterectomy for women with HMB/AUB by Solnik and Munro reports a significant difference in early postoperative complications between hysteroscopic surgeries and hysterectomy with 4/110 in hysteroscopic surgeries while 20/118 cases of hysterectomy with RR of 0.21 [CI 0.08, 0.61] [23]. It is similar in a study having 3/89 for TCRE and 4/92 for hysterectomy with significantly less RR of 0.78 [0.18, 3.37] for hysteroscopic procedure as reported by Zupi et al. for early complications of these two procedures [24].

In a study done by Selvanathan et al., a significant number of women who underwent hysterectomy had late and delayed adverse events at the end of one year when compared to hysteroscopy suggesting hysteroscopic surgical modality is found safer even when women are followed up for one year. They further state women who underwent hysterectomy had a higher occurrence of pelvic pain, bladder symptoms, sexual discomfort, and menopausal symptoms and they lasted for a longer duration of time when compared to hysteroscopic therapy [1].

Quality of Life (QoL)

Matteson et al. analyzed six studies that evaluated QoL and concluded that both the modalities had improvement. They further suggested that there is a need to study the long-term effects on QoL to compare the two surgical modalities [14]. Similar findings were seen in a review done by Zupi et al. [24].

In a study by Acharya et al., in both groups, viz. hysteroscopic therapy and hysterectomy group, the mean scores in all domains increased progressively at one week, six months, and one-year follow-up, but the hysteroscopy group had significantly better scores and improved rapidly and earlier when compared to those of hysterectomy group at all set of postoperative follow-up visits and resumed work early [1].

Abbott et al. studied quality of life as the primary outcome in terms of treatment results of endometrial ablation and concluded quality of life of women suffering from HMB improved with hysteroscopic ablation and it is a successful method to treat these women [25]. Table 1 given below summarizes the analysis of 25 articles.

**Table 1:** Analysis of the studies. AUB: Abnormal uterine bleeding; TVS: Transvaginal sonography; QoL: Quality of Life.

Sr no.	Author's name and reference numbers	Number of participants	Type of study/intervention	Conclusion
1	Acharya et al. [1]	354	Observational study - two groups. Group I – Hysteroscopic surgical procedure – polypectomy, endometrial resection, myomectomy. Group II – Hysterectomy – abdominal hysterectomy, vaginal hysterectomy, laparoscopic hysterectomy.	Both short-term and long-term QoL were better in the hysteroscopy group than hysterectomy.
2	Munro et al. [2]	-	(FIGO) International Menstrual Disorder Committee meets consensus for revision of basics of AUB terminologies and management option.	Clarifies terminologies related to AUB -classification types diagnosis and management options.
3	Vitale et al. [3]	111 studies	Review article	The individualized approach improves QoL with proper treatment options discussed with the patient.
4	Goldstein and Lumsden [4]	37 studies	Review article	Operative hysteroscopy is effective in preventing and postponing hysterectomy.
5	Whitaker and Critchley [5]	56 studies	Review article	Individualized and conservative treatment approach for AUB has better QoL scores for these women.
6	Medical Advisory Secretariat [6]	43 studies	Systematic review	Almost 15-20% of the perimenopausal age group suffer from AUB.
7	Goyal et al. [7]	100 patients	Interventional study	TVS is the first non-invasive investigation recommended for the diagnosis of the cause of AUB.
8	Bingol et al. [8]	346 patients	Interventional study	Saline infusion Sonosalpingography is superior to VS for the diagnosis of AUB.
9	Abid et al. [9]	241 patients	Cross-sectional study	Endometrial polyp being the most common cause of AUB, a conservative approach should be opted.
10	Pai et al. [10]	-	Narrative Review article	The review describes the diagnostic algorithm and focuses on conservative modalities for AUB management.
11	Fergusson et al. [11]	11 RCT	Cochrane Review	Endometrial resection and ablation offer an alternative to hysterectomy as a surgical treatment for heavy menstrual bleeding.
12	Abbott et al. [12]	37 patients	Interventional study	Uterine artery embolization further studies are recommended before considering it effective and safe for the treatment of AUB.
13	Lethaby et al. [13]	18 studies	Cochrane systematic review	Endometrial ablation techniques are effective in the management of AUB.
14	Matteson et al. [14]	7 studies	Narrative Review article	Hysteroscopic polypectomy should be followed by endometrial resection for symptom relief of AUB.
15	Stepniewska et al. [15]	60 patients	Interventional study	Radiofrequency thermal ablation as conservative approach can avoid hysterectomy in case of adenomyosis.
16	Piecak and Milart [16]	8 studies	Review article	Hysteroscopic removal with bipolar energy is effective for submucous myoma removal.
17	Agdi and Tulandi [17]	51 studies	Review article	Large number of myomas can be treated endoscopically and thereby conservatively.
18	Camanni et al. [18]	33 patients	Interventional study	Hysteroscopic myomectomy is effective when myoma is 6 cm or less in size.
19	Lasmar et al. [19]	191 patients	Multicentre prospective study	STEPW classification allows better prediction of success rate of myoma removal.
20	Donnez et al. [20]	-	Narrative review article	Hysetroscopic myoma resection is an effective conservative treatment option for submucous myomas.
21	van Dongen et al. [21]	60 patients	Randomised controlled trial	Hysteroscopic morcellation offers a good substitute to usual resection method.
22	Abdollahi Fard et al. [22]	277 patients	Descriptive cross-sectional	Hysteroscopy is a safe, precise and effective modality for management of AUB
23	Solnik and Munro [23]	-	Narrative Review article	Alternative therapies should be sought before opting for hysterectomy for women having AUB in the perimenopausal age group.
24	Zupi et al. [24]	181 patients	Interventional study	Being a definitive treatment and being associated with short-term and long-term effects on QoL are the pros and cons of hysterectomy when done for AUB.
25	Abbott et al. [25]	131 patients	Interventional study	Quality of life for women improves with a conservative hysteroscopic approach for AUB management.

## Conclusions

Heavy menstrual bleeding is a common cause of gynecologic consultation with a 24% incidence of overall gynecologic consultations. Hysteroscopic targeted therapy is effective in controlling menstrual bleeding in cases of AUB/HMB. It is also effective in relieving burdensome menstrual symptoms as a chief complaint in AUB/HMB. Hysteroscopic therapeutic procedures establish eumenorrhea or hypomenorrhea and amenorrhea in most of the patients at short-term and long-term follow-up which is a desirable effect with the benefit of conserving the uterus and avoiding hysterectomy. Those few cases who have persistent menstrual symptoms have a fair chance of responding to medical therapy as the primary cause is removed by hysteroscopy. Hence hysteroscopic therapeutic procedures are comparable in terms of effectiveness in relieving primary complaints of menstrual abnormality for women suffering from AUB/HMB and having the potential benefit of avoiding major and radical surgical procedures of hysterectomy.

Hysteroscopic therapeutic procedures are safer having reduced relative risk of intraoperative, early, late, and delayed postoperative complications as a surgical modality for the management of AUB/HMB when compared to hysterectomy. Hysteroscopic surgeries also have the benefit of less postoperative pain, shorter operation time, and hospital stay thereby having the benefit of early postoperative recovery and avoiding loss of working hours when compared to hysterectomy. Women who undergo hysterectomy, have significantly more risk of pelvic pain, bladder dysfunction, sexual dysfunction, and menopausal symptoms which last for a longer duration of time when compared to hysteroscopic therapy. Patients suffering from AUB/HMB have poor health-related quality of life (QoL). Both hysteroscopic therapy and hysterectomy as therapeutic modalities for HMB improve all domains of quality of life. The improvement in QoL in all domains is rapid and earlier in hysteroscopic therapy than that observed in the hysterectomy group.
